# *Mycobacterium smegmatis* GlnR Regulates the Glyoxylate Cycle and the Methylcitrate Cycle on Fatty Acid Metabolism by Repressing *icl* Transcription

**DOI:** 10.3389/fmicb.2021.603835

**Published:** 2021-02-03

**Authors:** Nan Qi, Guo-Lan She, Wei Du, Bang-Ce Ye

**Affiliations:** ^1^Institute of Engineering Biology and Health, Collaborative Innovation Center of Yangtze River Delta Region Green Pharmaceuticals, College of Pharmaceutical Sciences, Zhejiang University of Technology, Hangzhou, China; ^2^Lab of Biosystems and Microanalysis, State Key Laboratory of Bioreactor Engineering, Institute of Engineering Biology and Health, East China University of Science and Technology, Shanghai, China

**Keywords:** *Mycobacterium*, fatty acid metabolism, glyoxylate cycle, methylcitrate cycle, transcriptional regulation

## Abstract

*Mycobacterium smegmatis* (*Msm*), along with its pathogenic counterpart *Mycobacterium tuberculosis* (*Mtb*), utilizes fatty acids and cholesterol as important carbon and energy sources during the persistence within host cells. As a dual-functional enzyme in the glyoxylate cycle and the methylcitrate cycle, isocitrate lyase (ICL, encoded by *icl* or *MSMEG_0911*) is indispensable for the growth of *Msm* and *Mtb* on short-chain fatty acids. However, regulation of *icl* in mycobacteria in response to nutrient availability remains largely unknown. Here, we report that the global nitrogen metabolism regulator GlnR represses *icl* expression by binding to an atypical binding motif in the *icl* promoter region under nitrogen-limiting conditions. We further show that GlnR competes with PrpR, a transcriptional activator of *icl*, and dominantly occupies the co-binding motif in the *icl* promoter region. In the absence of GlnR or in response to the excess nitrogen condition, *Msm* cells elongate and exhibit robust growth on short-chain fatty acids due to the PrpR-mediated activation of *icl*, thereby inducing enhanced apoptosis in infected macrophages. Taken together, our findings reveal the GlnR-mediated repression of *icl* on fatty acid metabolism, which might be a general strategy of nutrient sensing and environmental adaptation employed by mycobacteria.

## Introduction

*Mycobacterium tuberculosis* (*Mtb*) is the pathogenic agent of tuberculosis (TB) that causes the death of millions of people annually (Data from the WHO Global Tuberculosis Report). Due to the long latency period, high pathogenicity, and the emergence of multidrug-resistant *Mtb* strains (Lin and Flynn, 2010; Getahun et al., 2015), seeking more effective TB treatments is a challenging task for medical researchers. Recently, studies have been focusing on microbial nutrient sensing and metabolism to explore their physiological significance in *Mtb* lifecycle and pathogenesis. It was reported that the metabolic enzymes of *Mtb*, such as methylisocitrate lyase (MCL) and malate synthase (MA), are important for its pathogenicity and survival (Eoh and Rhee, 2014; Puckett et al., 2017). Studies of the metabolic network in *Mtb* can help us to screen potential drug targets for TB treatment.

It is commonly accepted that *Mtb* utilize host-derived fatty acids as a important carbon source for bacterial growth and virulence (Munoz-Elias et al., 2006; Lovewell et al., 2016). Fatty acids are converted to acetyl-CoA (for even- and odd-chain fatty acids) and propionyl-CoA (for odd-chain fatty acids) by β-oxidation. Different enzymatic systems are involved in the conversion of acetate (an even-chain fatty acid) and propionate (an odd-chain fatty acid) to their CoA forms, acetyl-CoA and propionyl-CoA. Subsequently, acetyl-CoA is directed into the glyoxylate cycle, while propionyl-CoA is cyclically metabolized through the methylcitrate cycle in order to avoid the accumulation of toxic metabolic by-products (Munoz-Elias et al., 2006; Upton and McKinney, 2007). The first-step reaction of the glyoxylate cycle is catalyzed by the *icl*-encoded isocitrate lyase (ICL), which converts isocitrate into glyoxylate and succinate (Zu et al., 1999). In addition, ICL catalyzes another reaction producing succinate in the methylcitrate cycle, by using 2-methylisocitrate as the substrate. ICL thus plays a dual-role in the glyoxylate cycle and methylcitrate cycle essential for fatty acid metabolism in mycobacteria (Gould et al., 2006). The inability of *icl*-deficient *Mtb* to establish an infection in mice highlights the significance of ICL for pathogenesis (Munoz-Elias and McKinney, 2005). Recent studies have analyzed crystal structure of *Mtb* ICL and explored the potential of ICL as a target for the treatment of latent TB (Bhusal et al., 2017). Therefore, it is of great importance to investigate the regulation of *icl* and identify potential ICL inhibitors.

As a global response regulator, GlnR controls the transcription of genes involved in nitrogen and carbon metabolism for sensing different nutritional status in mycobacteria and other actinobacteria (Tiffert et al., 2008; Liao et al., 2015). For instance, genes encoding enzymes for ammonium assimilation (*glnII*, *gdhA*), nitrite reduction (*nirB*) and urea cleavage (*ureA*) are under transcriptional regulation by GlnR in *Streptomyces-coelicolor* (Tiffert et al., 2008; Pullan et al., 2011). Our previous studies have shown that GlnR regulates the methylcitrate cycle by inhibiting the transcription of *prpDBC* operon (*prpD* encode Methylcitrate dehydratase; *prpB* encode methylisocitrate lyase and *prpC* encode methylcitrate synthase) in *Mycobacterium smegmatis* (*Msm*), the non-pathogenic counterpart of *Mtb* (Liu et al., 2019). PrpR, a transcriptional activator of *icl1*, binds to the *prpDBC* promoter along with GlnR in *Msm* and *Mtb* (Paweł et al., 2012; Liu et al., 2019). However, whether *icl* is regulated by GlnR remains unknown. It is thus imperative to study if GlnR and PrpR co-regulate *icl*.

Here, we report the GlnR-mediated regulation of *icl* (also designed as *aceA* or *MSMEG_0911*) during *Msm* growth on fatty acids. We demonstrate that GlnR represses the transcription of *icl* and decreases the abundance of ICL products in the glyoxylate cycle and the methylcitrate cycle under nitrogen starvation condition, thereby impairing the growth, morphology and survival of *Msm* on short-chain fatty acids. Moreover, we unveil a competitive regulation of *icl* mediated by GlnR and PrpR, showing that GlnR blocks the PrpR-mediated activation of *icl* by occupying the co-binding motif in the promoter region. Our findings thus reveal a regulatory role of GlnR on fatty acid metabolism by repressing *icl*, which might be a general strategy employed by mycobacteria in response to different nutrition status.

## Results

### GlnR Represses the Transcription of *icl* in *Msm*

**FIGURE 1:**
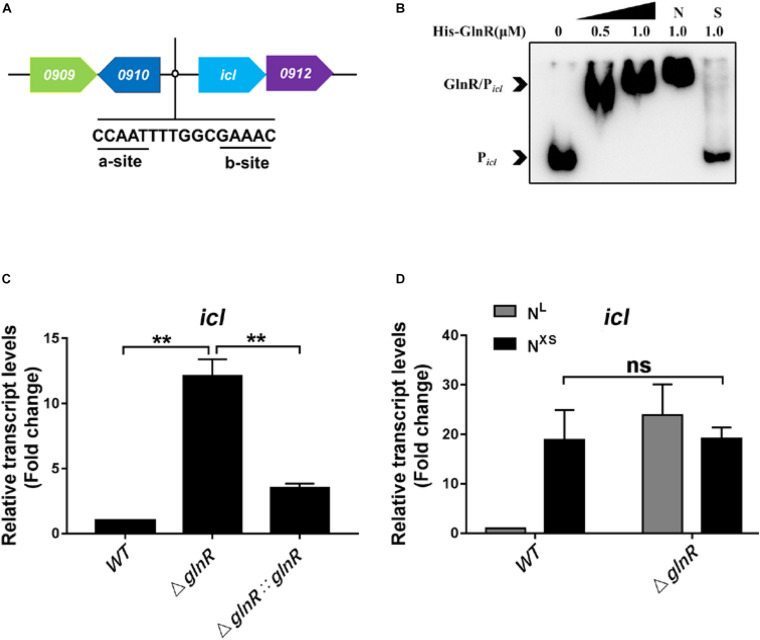
GlnR binds to the promoter region and represses the transcription of *icl* in *Msm.* **(A)** A diagram illustrating an atypical GlnR-binding motif in the *icl* (*MSMEG_0911*) promoter region of *Msm*. **(B)** EMSA showing the binding of recombinant GlnR with the upstream promoter region of *icl*. The DNA probe P*_icl_* (2 ng in a 10 μL reaction system) was incubated with a concentration gradient (0, 0.25, 0.5, and 1.0 μM) of His-tagged GlnR. S represents the unlabeled specific probe, N represents the non-specific competitor DNA (Salmon sperm DNA). **(C)** The transcript levels of *icl* in wild type (WT), Δ*glnR* and Δ*glnR:glnR* strains of *Msm* under nitrogen starvation. Diverse *Msm* were cultivated in the nitrogen-limited (N^L^) media and collected at the log phase. qRT-PCR was performed to determine the relative transcript fold (i.e., mutant vs. WT) of *icl* using the 2^– ΔΔCt^ method. The housekeeping gene *sigA* was used for normalization the transcript levels. **(D)** The transcription levels of *icl* in WT and Δ*glnR* of *Msm* in response to diverse nitrogen availability. The *Msm* wild type and Δ*glnR* strains were cultivated in the nitrogen-limited (N^L^) media or the nitrogen excess (N^XS^) media. qRT-PCR analysis was performed as described in panel **(C)**. Data are presented as mean values with error bars indicating standard deviations (±SD) calculated from three independent experiments. Unpaired two-tailed Student’s *t* test, **P < 0.01, ns indicates no statistically significant difference.

ICL is a dual-functional enzyme in the glyoxylate cycle and the methylcitrate cycle essential for the metabolism of fatty acids in *Msm* (Supplementary Figure S1). To study the regulation of *icl* by GlnR, we firstly searched for the GlnR-binding site in the promoter of *icl*. The classical binding framework of GlnR consists of two sites, a-site and b-site, separated by a hex-nucleotide motif (a-n6-b) (Fink et al., 2002; Tiffert et al., 2008; Liao et al., 2014). By sequence analysis using the Kyoto Encyclopedia of Genes and Genomes (KEGG) database and the Prokaryotic Operon database (ProOpDB)^1^, we identified a putative binding site of GlnR (GlnR-box), -CCAATTTTGGCGAAAC- at 300 bp upstream of the *icl* operon (Figure 1A). To further validate that GlnR could bind to the *icl* promoter region in *Msm*, recombinant His-tagged GlnR and biotin-labeled DNA probes containing the putative GlnR-box were subjected to the electrophoretic mobility shift assay (EMSA). Unlabeled specific probe (S) (200-fold excess) and non-specific competitor DNA (Salmon sperm DNA) (N) were used as controls. As shown in the Figure 1B, obvious shift bands were detected following the incubation of recombinant GlnR with the specific DNA probes, demonstrating that GlnR directly binds to the *icl* promoter region in *Msm*. To further validate the binding activity, we introduced loss-of-function mutations into the GlnR-binding motif as previously described (Jenkins et al., 2013). Mutation in the typical b-site (replacing -GAA**AC**- with -GAA**GG**-) completely abolished the binding of GlnR to the *icl* promoter (Supplementary Figure S2A). Notably, the a-site mutation (replacing -CCA**AT**- with -CCA**GG**-) in GlnR-box substantially attenuated the binding affinity of GlnR to the *icl* promoter, as we observed a faster electrophoretic mobility of GlnR-P*_icl_* which is attributed to the less protein binding (Muller and Salles, 1997; Pham et al., 2004; Xu et al., 2019). These results suggest that both the typical b-site and the atypical a-site in GlnR-box of *icl* promoter are indispensable for GlnR binding. Furthermore, we detected a weak binding affinity between GlnR and the *Mtb icl* promoter (Supplementary Figures S2B,C), suggesting that the atypical GlnR-box might be conserved in the *icl* promoter in mycobacteria.

We next examined whether GlnR directly regulates the transcription of *icl* on fatty acid metabolism. To this end, *Msm* were cultured in minimal medium with propionate as the sole carbon source. Since the expression of *glnR* responses to the nitrogen starvation (Amon et al., 2008; Jenkins et al., 2013), we analyzed the differential transcription levels of *icl* among *Msm* wild type strain (WT), the *glnR*-deficient strain (Δ*glnR*) described previously (Liu et al., 2019), and the *glnR* complemented strain (Δ*glnR:glnR*) under the nitrogen-limited (N^L^, 1 mM ammonium sulfate) condition. Compared with WT *Msm*, the Δ*glnR* strain exhibited an increased *icl* transcript level, which returned to basal levels upon complementation by *glnR* (Figure 1C). It has been well-established that the *glnR* expression in WT *Msm* is completely blocked in response to the excess nitrogen (N^XS^, 30 mM ammonium sulfate) condition (Xu et al., 2017). Under the N^XS^ condition, *icl* transcript levels dramatically increased in WT *Msm*, to reach the same levels as in the Δ*glnR* strain (Figure 1D), further demonstrating that the down regulation of *icl* transcription is mediated by GlnR. Under the same conditions, GlnR had no impacts on the transcription of another ICL-encoding gene *MSMEG_3706* (Supplementary Figure S2D). Moreover, the repression of *icl* transcription by GlnR was validated when the carbon source propionate was replaced with other short-chain fatty acids like acetate, butyrate and valerate (Supplementary Figure S2E). We thus conclude that GlnR is a transcriptional repressor of *icl* (*MSMEG_0911*) during *Msm* growth on short-chain fatty acids.

### GlnR Blocks the PrpR-Mediated Activation of *icl*

**FIGURE 2:**
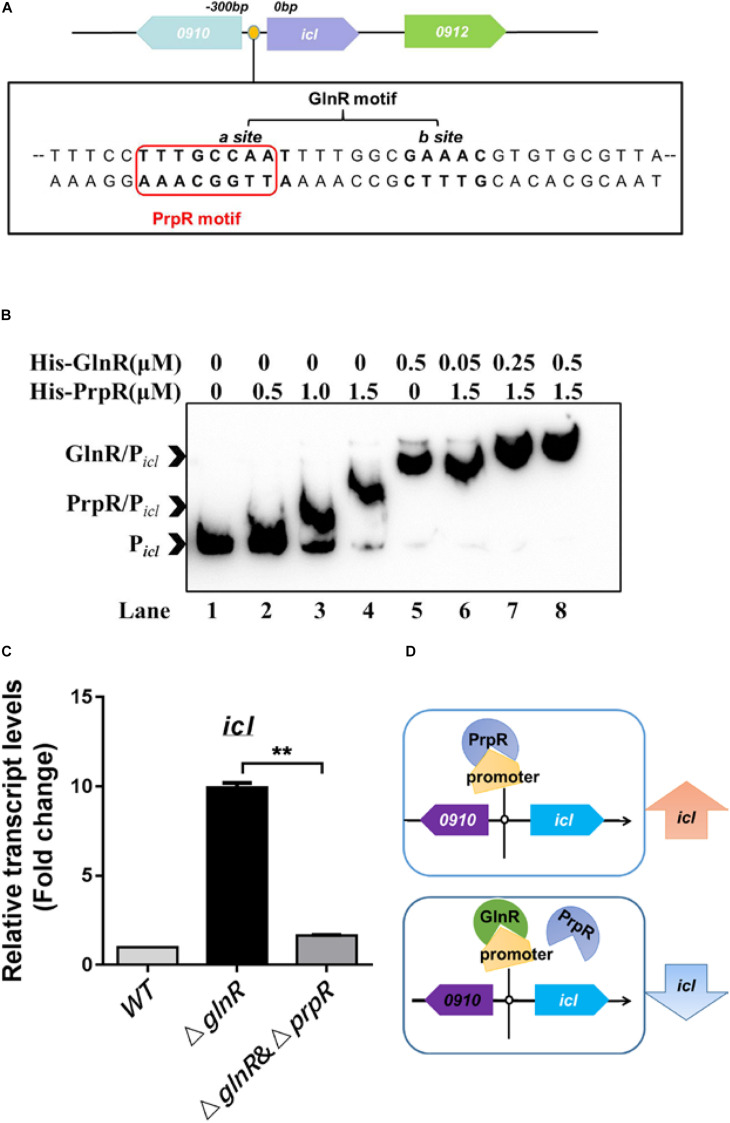
GlnR and PrpR competitively regulate the *icl* transcription under the nitrogen limited conditions. **(A)** A diagram illustrating the co-binding motif of GlnR (-CCAAT-n6-GAAAC-) and PrpR (-TTTGCAAA-) in the *icl* promoter region of *Msm*. **(B)** Competitive EMSA of P*_icl_* with GlnR and PrpR. Lane 1, probe without proteins; Lane 2 to 4, increasing concentrations of PrpR protein (from 0.5 to 1.5 μM); Lane 5, probe with GlnR protein only; Lane 6 to 8, increasing concentrations of GlnR protein (from 0.05 to 0.5 μM) mixed with PrpR protein at a constant concentration of 1.5 μM. **(C)** qRT-PCR analysis of the transcriptional levels of *icl* in WT, Δ*glnR* and Δ*glnR*Δ*prpR* strains of *Msm*. WT, Δ*glnR* and Δ*glnR*Δ*prpR Msm* strains were cultivated in the nitrogen-limited medium, and collected at the log stage. RNA were extracted and then subject to qRT-PCR. Data are presented as mean values with error bars indicating standard deviations (±SD) calculated from three independent experiments. Unpaired two-tailed Student’s *t* test, **P < 0.01, ns indicates no statistically significant difference. **(D)** A proposed model of the GlnR and PrpR-mediated competitive regulation of *icl*. Under the nitrogen starvation, GlnR dominantly occupies the co-binding motif in the *icl* promoter region, thus blocking the PrpR-mediated activation of *icl*. In the absence of GlnR or response to the excess nitrogen conditions, PrpR binds to the promoter region and activates the transcription of *icl*.

It was previously reported that PrpR activates *icl1* transcription in *Mtb* and *Msm* (Paweł et al., 2012). In the *icl* promoter region, we noticed that a typical binding motif of PrpR (PrpR-box), - TTTGCAAA-, is present in the GlnR-box (Figure 2A). All these findings inspired us to speculate that GlnR and PrpR coordinately regulate *icl* transcription in the methylcitrate cycle. To test this hypothesis, we performed competitive EMSA to examine whether GlnR and PrpR are competing with each other to bind to the probe P*_icl_*, which is derived from the *icl* promoter containing both GlnR- and PrpR-box. As shown in Figure 2B, recombinant PrpR bound to P*_icl_*, while an increasing dose of GlnR shifted the mobility of PrpR/P*_icl_* binding band to that of GlnR/P*_icl_*, indicating that GlnR dominates PrpR in competing for the *icl* promoter. In addition, we measured the affinity constants (KD values) of GlnR and PrpR for P*_icl_*. The KD value of PrpR for P*_icl_* is nearly 5-fold higher than the KD value of GlnR for P*_icl_* (Supplementary Figures S3A,B), further demonstrating that GlnR has higher affinity for the *icl* promoter than PrpR.

To study the competitive effects of GlnR with PrpR on regulating *icl* transcription, we deleted *prpR* in the genome of the *Msm* Δ*glnR* strain, thereby generating the Δ*glnR*Δ*prpR* strain. We found that the transcriptional induction of *icl* caused by *glnR*-deficiency is attributed to the PrpR-mediated activation, as the increased mRNA level of *icl* in the Δ*glnR* strain was returned to basal level in the Δ*glnR*Δ*prpR* strain (Figure 2C, compare Δ*glnR* and Δ*glnR*Δ*prpR*). As illustrated in our proposed model (Figure 2D), these collective findings suggest that GlnR has a higher affinity to the shared region of *icl* promoter in comparison with PrpR, thus antagonizing the PrpR-mediated activation of *icl*.

### GlnR Decreases the Cellular Concentrations of Intermediates in the Glyoxylate Cycle and the Methylcitrate Cycle During *Msm* Growth on Propionate

**FIGURE 3:**
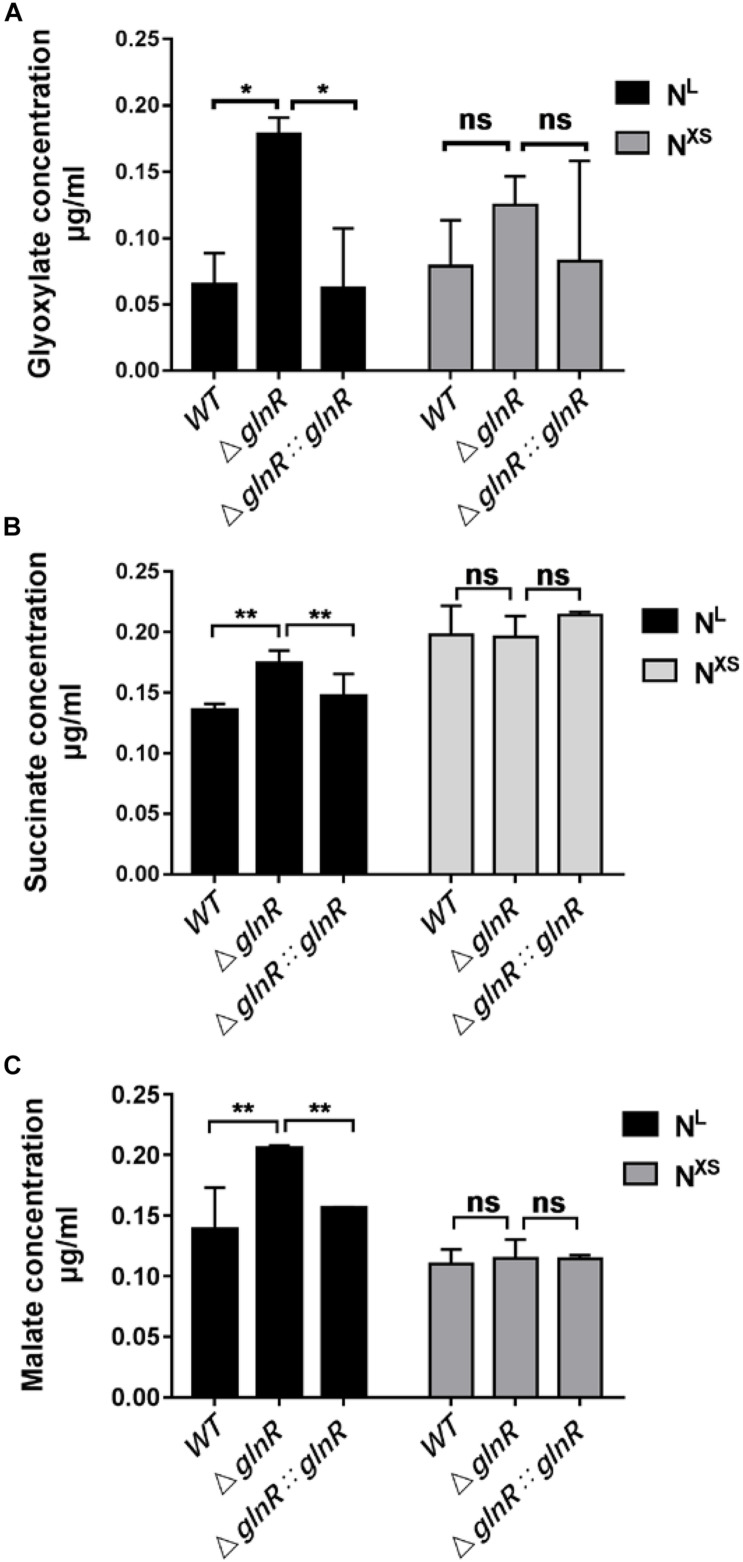
The Δ*glnR* strain accumulates higher concentrations of intermediates in the glyoxylate cycle and the methylcitrate cycle. Under the N^L^ or the N^XS^ growth conditions, *Msm* WT, Δ*glnR* and Δ*glnR::glnR* strains were collected to quantify the levels of glyoxylate **(A)**, succinate **(B)** and malate **(C)** by LC-MS. N^XS^ means the nitrogen excess and N^L^ represents the nitrogen limitation, respectively. Data are presented as mean values with error bars indicating standard deviations (± SD) calculated from three independent experiments. Unpaired two-tailed Student’s *t* test, **P < 0.01, ns indicates no statistically significant difference.

Given that the *icl* transcription is repressed by GlnR under nitrogen starvation, it is of interest to examine whether GlnR impairs the *icl*-encoded ICL enzyme activities. We thus used LC-MS to quantify the differential levels of ICL products in the glyoxylate and the methylcitrate cycles among diverse *Msm* strains during growth on propionate. The cellular concentrations of ICL-catalyzed products, glyoxylate and succinate, as well as the downstream intermediate malate, were increased in the Δ*glnR* strain during growth on propionate under the nitrogen-limited conditions (Figures 3A–C). However, this pronounced effect induced by the *glnR-*deletion was not detected under the excess nitrogen culture conditions. Together with the transcriptional analysis, these data suggest that the repression of *icl* expression by GlnR decreases the flux of ICL-catalyzed products through the glyoxylate cycle and methylcitrate cycle.

### GlnR Hinders the Growth and Cell Elongation of *Msm* on Short-Chain Fatty Acids

**FIGURE 4:**
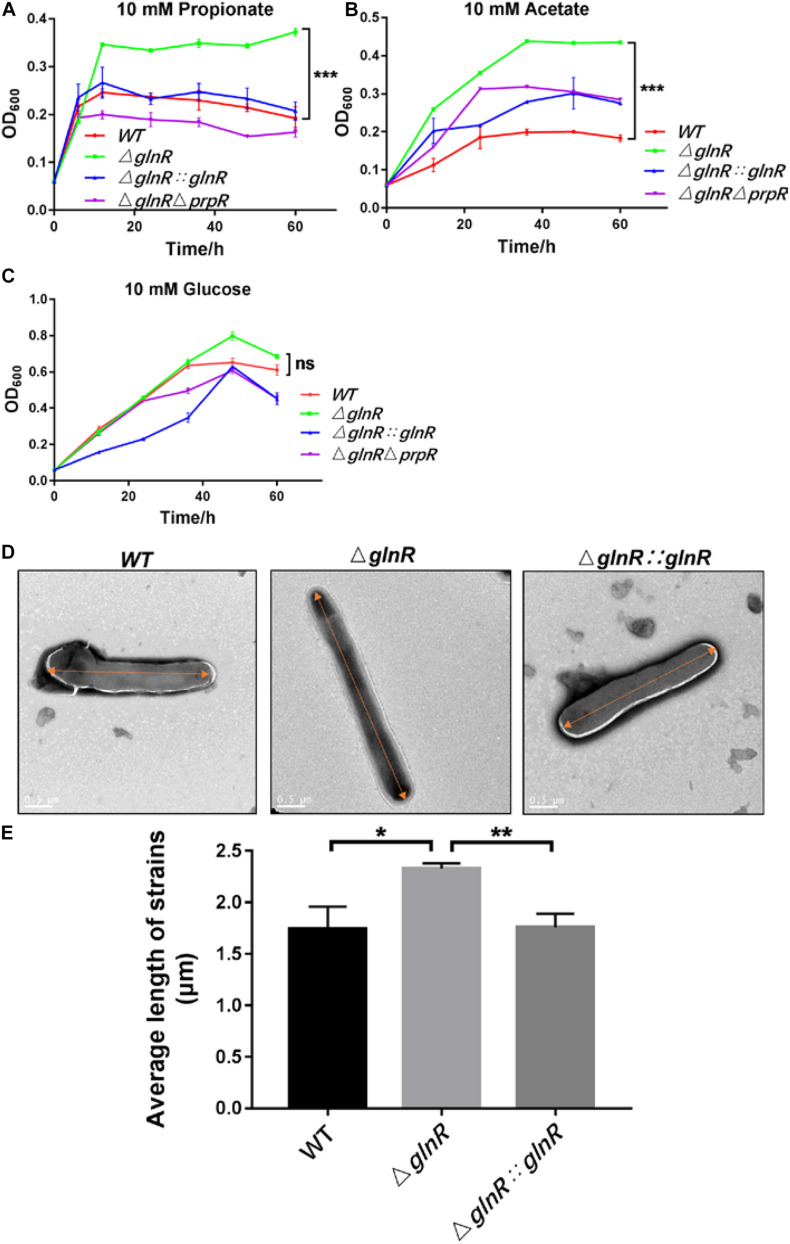
The Δ*glnR* strain grows to higher optical densities and grows into elongated cells in response to short-chain fatty acids. The growth curves of *Msm* WT, Δ*glnR* and Δ*glnR*Δ*prpR* strains growing in minimal medium with 10 mM propionate **(A)**, acetate **(B)**, glucose **(C)**, respectively. Error bars represent the standard deviations from three biological replicates. **(D,E)** Deletion of *glnR* elongates bacteria cells during *Msm* growth on propionate. Scanning electron microscopy (SEM) images of bacteria cells from *Msm* WT, Δ*glnR* and Δ*glnR*Δ*prpR* strains **(D)**. Scale bar represents 0.5 μm. Length of bacteria cells (*n* = 50) was measured by manual evaluation, using Image J on the electron microscopic images **(E)**. Data are presented as mean values with error bars indicating standard deviations (±SD) calculated from three independent experiments. Unpaired two-tailed Student’s *t* test, *P < 0.05, **P < 0.01, ***P < 0.001, ns indicates no statistically significant difference.

Having demonstrated that GlnR likely decreases flux through the glyoxylate cycle and methylcitrate cycle by transcriptionally repressing the expression of *icl*, we next studied the physiological effects of GlnR-mediated down-regulation on *Msm* growth. For this purpose, the growth curves of various *Msm* strains were determined under diverse culture conditions. It was shown that the Δ*glnR* strain grows to higher final cell densities when using propionate or acetate as the sole carbon source, while this pronounced effect on growth were diminished by complementing with *glnR* or deleting *prpR* (Figures 4A,B). When glucose was the sole carbon source, no significant differences were observed among the growth curves of each strain (Figure 4C). We further observed the morphology of various *Msm* strains using scanning electron microscopy. Fifty cells were measured and calculated the average value for each strain. The average length of WT was 1.74 ± 0.22 μm; the (glnR strain was 2.33 ± 0.05 μm and (glnR:::glnR strain was 1.76 ± 0.13 μm. Notably, the glnR-deficiency resulted in an elongation of Msm cells when growing in propionate, as the cell length of (glnR strain were significantly longer than that of WT and the (glnR:::glnR strain (Figures 4D,E and Supplementary Figure S4). An explanation is that the morphological elongation facilitates the Δ*glnR* strain to more efficiently absorb nutrients and grow better on the short-chain fatty acids. These findings thus establish GlnR as a negative effector on the growth of *Msm* in response to propionate.

### Deletion of *glnR* Enhances the Apoptosis of Macrophages Infected With *Msm*

**FIGURE 5:**
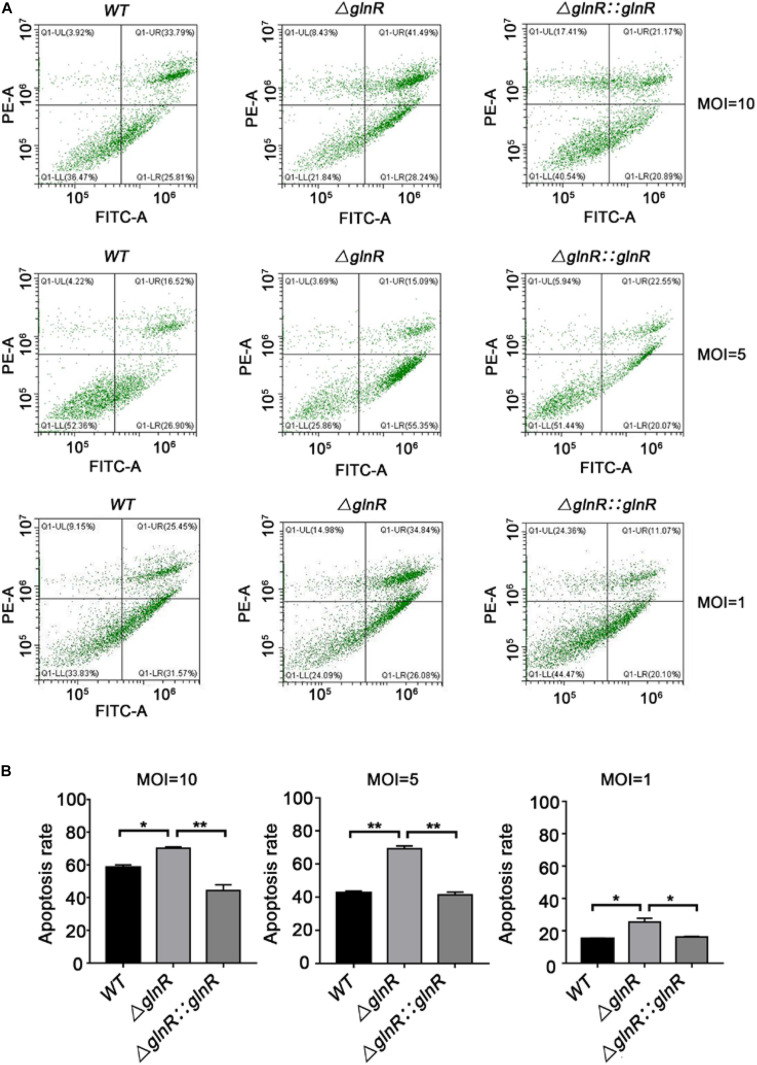
Strain Δ*glnR* of Msm enhances apoptosis in infected macrophages. THP-1 derived macrophages were infected with mid-logarithmic (OD_600_ = 0.4) *Msm* WT, Δ*glnR*, and Δ*glnR*::*glnR* strains at a MOI of 10, 5, or 1. At 72 h post-infection, analysis of cell apoptosis was conducted by flow cytometry (FCM). **(A)** The effects of *Msm* WT, Δ*glnR* and Δ*glnR*::*glnR* strains on cell apoptosis were determined through the FCM. Lower right represents the apoptotic cells and upper right represents the necrotic cells. **(B)** A histogram calculating the apoptosis rate of macrophages infected with various *Msm* strains as described in panel **(A)**. Data are presented as mean values with error bars indicating standard deviations (±SD) calculated from three independent experiments. Unpaired two-tailed Student’s *t* test, *P < 0.05, ***P < 0.001, ns indicates no statistically significant difference.

**FIGURE 6:**
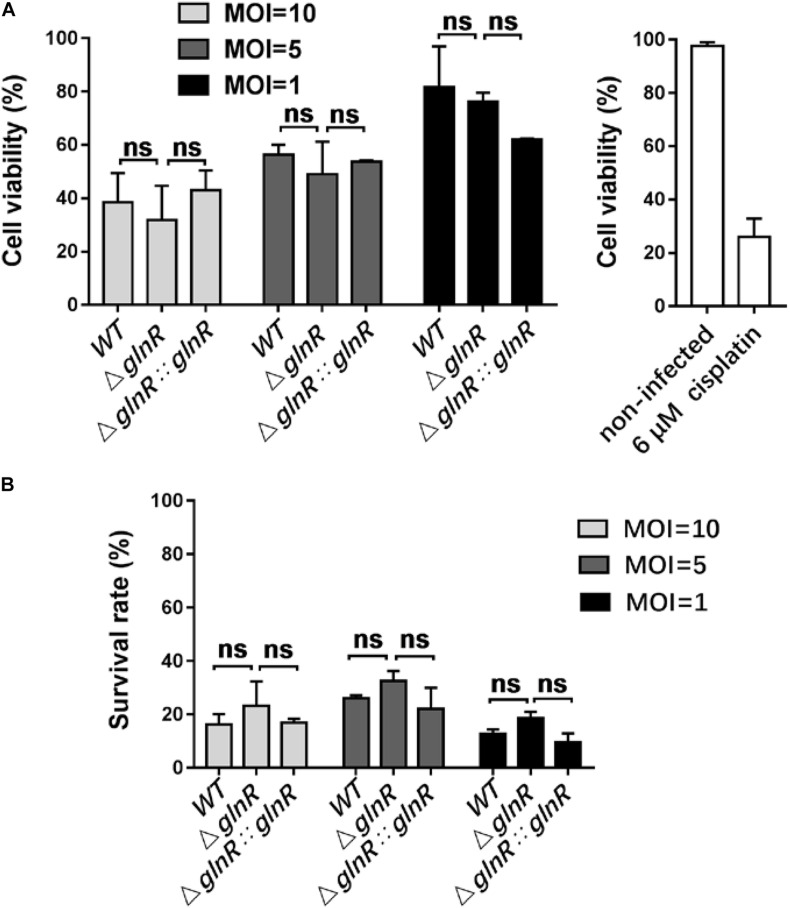
Deletion of *glnR* in *Msm* has no impacts on the proliferation of infected macrophages and the survival of bacteria in macrophages. **(A)** Cell proliferation and viability of macrophages infected with *Msm* WT, Δ*glnR*, and Δ*glnR*::*glnR* strains. Macrophages were infected with diverse *Msm* strains at MOI = 10, 5, 1, and subjected to MTT assay at 72 h post-infection. Cell viability (%) represents the ratio of OD_570_ value of infected cells to that of non-infected cells. Cisplatin (6 μM) was used as the positive control. **(B)** Survival of *Msm* WT, Δ*glnR*, and Δ*glnR:glnR* strains in macrophages. At 0 or 72 h post-infection, bacteria in macrophages were collected for measuring the value of OD_600_. Survival rate (%) represents the ratio of 72 h-OD_600_ value to 0 h-OD_600_ value. MOI = 10, 5, 1. Data are presented as mean values with error bars indicating standard deviations (±SD) calculated from three independent experiments. Unpaired two-tailed Student’s *t* test, ns indicates no statistically significant difference.

To study the physiological effects of GlnR-mediated regulation on *Msm* survival, THP-1-derived macrophages were infected with various *Msm* strains cultured on propionate. We used flow cytometry to measure the apoptosis rate of macrophages infected with mid-logarithmic (OD_600_ = 0.4) *Msm* at a multiplicity of infection (MOI) of 1, 5, or 10. To study if a release of metabolites from high amount of *Msm* would induce apoptosis, we performed comparison analysis between mutant and parental strains. Comparative experiments were also conducted using alive and killed bacteria. The Δ*glnR* strain consistently led to an enhanced apoptosis of *Msm*-infected macrophages at different MOI (Figures 5A,B). On the contrary, no difference was detected in the apoptosis rates of macrophages infected with killed bacteria (Supplementary Figure S5). These data indicate that the enhanced apoptosis induced by the Δ*glnR* strain is not related to the metabolites. Furthermore, we found that neither the proliferation of macrophages nor the survival of *Msm* in macrophages were impaired by deleting *glnR*, as no statistical difference was observed in the cell viability of macrophages or the survival rate of bacteria when comparing WT *Msm* and the Δ*glnR* strain (Figures 6A,B). Taken together, these results suggest that GlnR negatively impairs the apoptosis of macrophages infected with *Msm* which were pre-adapted to grow on propionate.

## Discussion

GlnR is a DNA-binding protein that regulates the transcription of genes related to nitrogen uptake and metabolism in both *Mtb* and *Msm* (Amon et al., 2008). Apart from nitrogen metabolism, GlnR also controls the expression of genes involved in carbon metabolism. Our previous work reported that *Msm* GlnR affects the methylcitrate cycle through regulating the *prpDBC* operon by binding to the conserved motif GGACC-GGCACC-GTAAC (Liu et al., 2019). In this paper, we identified an atypical GlnR-binding motif, CCAAT-n6-GAAAC, in the promoter region of *icl* in *Msm*, and revealed that GlnR represses *icl* transcription. Moreover, we identified a GlnR-binding motif in upstream of the *icl* promoter in *Mtb*, and verified the binding of GlnR to P*_icl_* by EMSA. Since *glnR* is highly conserved within the actinomycetes, future study is ongoing to test whether the GlnR-mediated regulation of *icl* is general in *Mtb* and other actinomycetes.

**FIGURE 7:**
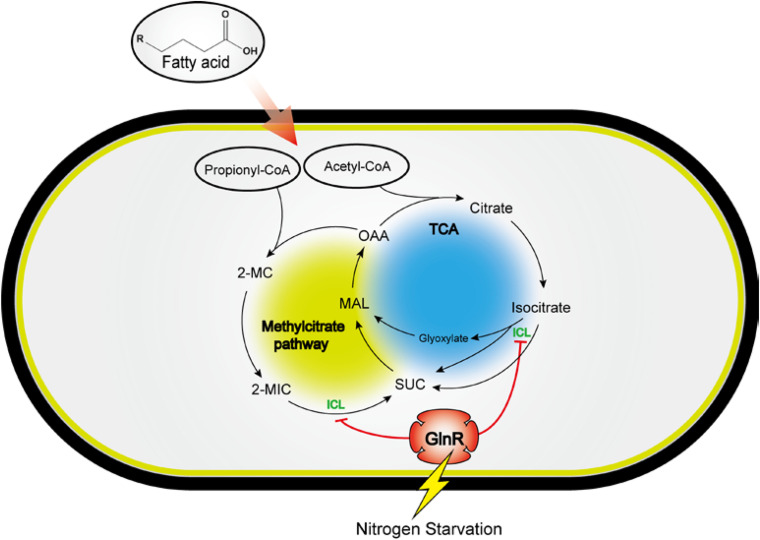
A model for the GlnR-mediated regulation of *icl* in fatty acid metabolism, revealing a tight connection of carbon metabolism and nitrogen sensing in *Msm*. Circle with blue: TCA cycle and glyoxylate cycle; circle with yellow: Methylcitrate cycle; SUC, succinate; MAL, malate; OAA, oxaloacetate; 2-MC, 2-methylcitrate; 2-MIC, 2-methylisocitrate; ICL, isocitrate lyase.

It was previously reported that PrpR directly activates the transcription of *icl1*, which encodes ICL involved in the glyoxylate cycle and methylcitrate pathways in both *Mtb* and *Msm* (Paweł et al., 2012). In our present work, PrpR is demonstrated to activate *icl* transcription in *Msm* once GlnR is absent or inactive under the excess nitrogen condition. A plausible explanation is that the higher binding affinity enables GlnR to occupy the promoter region of *icl* in competition with PrpR, thereby blocking the PrpR-mediated transcriptional activation of *icl*. We propose a model of the GlnR- and PrpR-mediated co-regulation of *icl* in fatty acid metabolism, which might be an adaptation strategy employed by mycobacteria in response to environmental nitrogen availability (Figure 7). In the evolutionary arms race between hosts and pathogens, host cells are able to antagonize *Mtb* by limiting the availability of amino acid such as aspartate, a major nitrogen source during *Mtb* infection (Shin et al., 2011; Zhang and Rubin, 2013). We speculate that *Mtb* adopts the GlnR-mediated regulatory mechanism to slow down its growth on host-derived fatty acid to adapt to the nitrogen-limited intracellular environment. At the late stage of infection, *Mtb* can synthesize its own amino acids and grow independently of the nitrogen-limited conditions in host cells (Zhang and Rubin, 2013). The nitrogen excess removes the GlnR-mediated repression of *icl* in *Mtb*, thus accelerating *Mtb* growth and enhancing the apoptosis of host cells. Therefore, our studies provide new insights into the host-pathogens interaction based on bacterial sensing and metabolism.

Recent studies have elucidated the carbon flux in *Mtb*, primarily focusing on those pathways responsible for the transformation of propionyl-CoA into intermediates of TCA cycle like succinate, malate and oxaloacetate (Savvi et al., 2008; Puckett et al., 2017). Detoxification of propionyl-CoA present on the operation of the methylcitrate cycle and the methylmalonyl cycle, or incorporation of the propionyl-CoA into methyl-branched lipids of host cells, enabling *Mtb* to utilize and produce energy for their pathogenesis (Lovewell et al., 2016; Puckett et al., 2017). Recent discoveries are establishing lactate and pyruvate as important energy and carbon sources for *Mtb*, and showing that they are assimilated by through glyoxylate shunt and methylcitrate cycle (Billig et al., 2017; Osada-Oka et al., 2019; Serafini et al., 2019). Interestingly, the methylcitrate cycle can operate in reverse as a pathway for the synthesis of propionyl-CoA. The fact that *Mtb* can use the methylcitrate and glyoxylate cycles to assimilate carbon sources different from beta-oxidation derived products suggests that GlnR might regulate these pathways independently from a certain carbon source including the short-chain fatty acids. In addition to the *icl* transcript levels, our present work also explored the physiological effects of GlnR-mediated regulation on *Msm* survival, morphology and pathogenesis. Our data showed that the *glnR*-deficiency in *Msm* enables bacteria to grow to higher final cell densities and trigger the cell elongation in response to short-chain fatty acids. The increased apoptosis of macrophages infected with *Msm* lacking GlnR was suspected to be connected to the role of GlnR in the regulation of certain metabolic pathways, as it is unknown which nutrient activates such pathways during infection. Here, we proposed two mechanistic hypotheses regarding why the Δ*glnR* strain has improved growth on propionate and was morphologically elongated in comparison with WT *Msm*: (1) Since the elevated ICL activities by *glnR*-deficiency accelerate the propionyl-CoA metabolism in the Δ*glnR* strain, the assimilation of propionyl-CoA and other toxic metabolites such as 2-methylcitrate are strengthened, posing a positive impact on the growth of *Msm* utilizing propionate as the sole carbon source. (2) Since the intermediate of propionyl-CoA metabolism, succinate, is also linked to methylmalonyl-CoA metabolism, we speculated that increase of succinate in the Δ*glnR* strain would probably enhance the metabolic flux of methylmalonyl-CoA, which is coupled with the synthesis of lipids (PDIM and SL-1) in bacterial cell wall (Kumar et al., 2007). Owing to the accelerating synthesis of lipids in cell wall, the bacteria cells of *Msm* Δ*glnR* strain are elongated. Since *Msm* is not a host-adapted bacterium, the nutrient response of *Msm* might be different from that of *Mtb* adapting to the host intracellular environment. Studies based on a *Mtb*-infected mice model is imperative to elucidate the nutrient response of *Mtb* in macrophages, and further explore the physiological significance in the future.

ICL has emerged as a potential drug target for TB therapy. Currently, inhibitors of ICL, such as 3-nitropropionate, block *Mtb* growth in infected murine macrophages (McFadden, 1977; Altaf et al., 2010). However, these chemicals are unsuitable for TB therapy due to their side effects inhibiting key metabolism enzymes activities of host. As revealed in our present work, GlnR is a repressor of *icl* and affects ICL production under the nitrogen-limited condition. Therefore, nutrient adjustment reducing nitrogen uptake might be a potential adjuvant treatment of TB, through the GlnR-mediated repression of ICL activities and *Mtb* persistence in host cells.

## Materials and Methods

### Strains, Plasmids and Growth Conditions

Strains and plasmids used in this work were listed in Table 1. *E. coli* strains were grown at 37°C in liquid or on solid LB medium. *Msm* wild type, Δ*glnR*, Δ*glnR*::*glnR* and Δ*glnR*Δ*prpR* strains were cultivated on liquid LB medium which contains 0.05% tween-80. After being washed for three times with normal saline, the bacteria were incubated in nitrogen free Sauton’s medium [0.05% (w/v) KH2PO4, 0.05% (w/v) MgSO4, 0.2% (w/v) citric acid, 0.005% (w/v) ferric citrate, 0.2% (v/v) glycerol, 0.0001% (v/v) ZnSO4, 0.015% (v/v) Tyloxapol] (Jenkins et al., 2013) with 1 mM ammonium sulfate (N^L^) or 30 mM ammonium sulfate (N^XS^) and 10 mM sodium propionate used for transcription analysis, scanning electron microscopy analysis and mass spectrometry analysis, respectively. *Msm* wild type, Δ*glnR*, Δ*glnR*::*glnR* and Δ*glnR*Δ*prpR* strains were grown in M9 medium (Hayden et al., 2013; Liu et al., 2019) containing 10 mM glucose, 10 mM sodium acetate or 10 mM sodium propionate to measure the growth curve. All the strains were grown at 37°C and the medium were sterilized by autoclaving at 121°C for 20 min.

**TABLE 1:** Strains and plasmids used in this study.

Strains/plasmids	Source or Reference
*E. coli* BL21(DE3)	Novagen
*E. coli* DH5α	Novagen
*Msm mc^2^ 155*	Dr. Brian D. Robertson gifted
*Msm mc^2^ 155 ΔglnR*	Dr. Brian D. Robertson gifted
*Msm mc^2^ 155 ΔglnRΔglnR*	Constructed by our laboratory (Liu et al., 2019)
pET-28a	Novagen
pMV361-*glnR*	Constructed by our laboratory
pET-28a-*prpR*	Constructed by our laboratory
pET-28a-*glnR*	Constructed by our laboratory
pET-28a-*Rv-glnR*	Constructed by our laboratory
*Msm mc^2^ 155 ΔglnRΔprpR*	In this work
*pPR27*	DipankarChatterji

### Construction of Deletion Mutant Strains

The gene knockout strategy was described in previous studies (Pelicic et al., 1997; Mathew et al., 2005). Briefly, a recombination cassette was constructed to delete prpR from the *Msm* Δ*glnR* chromosome. It consisted of a 1.3 kb DNA fragment on the upstream of *prpR* and a downstream fragment of DNA about 1.2 kb with the fragment holding the *kan* gene from plasmid PET-28a between them. After the preparative cloning steps, the whole recombination cassette was transferred to the suicide vector *pPR27* to get the final construct. The *sacB* mutant gentamicin-susceptible and kanamycin-resistant colonies were selected for further analysis. The selected mutants (Δ*glnR*Δ*prpR*) were verified by PCR and DNA sequencing. Sequences of primers were shown as in Table 2.

**TABLE 2:** List of primers used in this study.

Name	Sequence (5′→3′)	purpose
MSM6643- UP-F	GAATTGGAGCTCCACCGCGGTGGCGGCCGCCAGTTCGATGGTGTCCGGTCGGTGC	amplification of Msmeg_6643 upstream fragment
MSM6643- UP-R	CAAGACGTTTCCCGTTGAATATGGCTCATACACACAGCCTAACCGCGATTCTTACACAG	amplification of Msmeg_6643 upstream fragment
Kan-F	CTGTGTAAGAATCGCGGTTAGGCTGTGTGTATGAGCCATATTCAACGGGAAACGTCTTG	amplification of Kanamycin encodes genes
Kan-R	GGGCGGCACTGATCAGCGGGGGTCGTTGATTAGAAAAACTCATCGAGCATCAAATG	amplification of Kanamycin encodes genes
MSM6643- DOWN-F	CATTTGATGCTCGATGAGTTTTTCTAATCAACGACCCCCGCTGATCAGTGCCGCCC	amplification of Msmeg_6643 downstream fragment
MSM6643- DOWN-R	GTGAGGATCGGGGGATCCACTAGTTCTAGAGGCACTTCGGGTGTTCAAGGCCATC	amplification of Msmeg_6643 downstream fragment
MSM6643- DE-F	CCAGGGTGACGAACTTGTTGC	verify the deletion of Msmeg_6643
MSM6643- DE-R	CTCGCATCAACCAAACCGTTATTCA	verify the deletion of Msmeg_6643
Msmeg_0911_S	AGCCAGTGGCGATAAG ATATGCGGTCGGCGAGGAGAC	amplification of Msmeg_0911 upstream regions
Msmeg_0911_A	AGCCAGTGGCGATAAG GTTGTGATCCCAGTCGTGCTGGATC	amplification of Msmeg_0911 upstream regions
Universal primer	biotin-AGCCAGTGGCGATAAG	amplification of Msmeg_0911 upstream regions
MSM5784RT-F	GCGTAGATGGCTGCTCCGAAAT	used for Q-RT-PCR
MSM5784RT-R	CGCAGACTGGTGGTCGGGTT	used for Q-RT-PCR
MSM6643RT-F	CACCATCTCCTCGACATCGG	used for Q-RT-PCR
MSM6643RT-R	CAGGCTCTTTACGTGCCATTAG	used for Q-RT-PCR
MSM0911RT-F	CCTCGGAGAAGAAGTGCGGC	used for Q-RT-PCR
MSM0911RT-R	GAGGTCAGGGTGCGGATGTG	used for Q-RT-PCR

*Sequences of each primer in 5′ to 3′ are given. The purpose of each oligo in this study is also mentioned.*

### Protein Expression and Purification in *E. coli* BL21 (DE3)

The protein His-GlnR, His-PrpR and His-Rv-GlnR were expressed in *E. coli* BL21 (DE3) strains which was constructed in our previous work, strains were cultured in 5 ml LB (0.05 mg/ml kanamycin) overnight, and then transferred to 50 ml LB (0.05 mg/ml kanamycin). A total of 0.7 mM isopropyl-β-D- thiogalactopyranoside was added when the optical density at 600 nm (OD_600_) of the cells was about 0.6. Then, the cells were grown at 20°C overnight (Yao and Ye, 2016; Liu et al., 2019). The purified proteins were assessed by sodium dodecyl sulfate-polyacrylamide gel electrophoresis, and protein concentration was determined using the Bradford reagent.

### Electrophoretic Mobility Shift Assay (EMSA)

The upstream regions (−300 to +50) of *icl* (*MSMEG_0911*) was amplified by PCR using gene-specific primers containing the universal primer (5′-AGCCAGTGGCGATAAG-3′) sequence (Table 2) and biotin labeled by PCR using the 5′ biotin-modified universal primer. The PCR products were analyzed by agarose gel electrophoresis and purified using a gel extraction kit (Transgen Biotech Co., Ltd., Beijing, China). The concentration of biotin-labeled DNA probes was determined using a microplate reader (BioTek, United States). The next steps were the same as our previous work (Xu et al., 2016; Yao and Ye, 2016; Liu et al., 2019). EMSAs were carried out according to manufacturer protocol for the chemiluminescent EMSA kit (Beyotime Biotechnology, Jiangsu, China).

### Quantitative Real-Time PCR

Cells at exponential stage in nitrogen-limited medium were collected by centrifugation. Total RNA was prepared using an RNeasy mini kit (Qiagen, Valencia, CA, United States). The RNA quality was analyzed by 1% agarose gel electrophoresis, and the concentration was determined by microplate reader (BioTek, United States). The RNA was reverse transcribed to cDNA using a PrimeScript reverse transcription (RT) reagent kit with gDNA Eraser (TaKaRa, Shiga, Japan), and DNase digestion was performed to remove genomic DNA before reverse transcription for 5 min at 42°C. The RT-PCR was performed with primers listed in Table 2 and performed in a 20 μL PCR solution from the SYBR premix Ex Taq GC kit (Perfect Real Time; TaKaRa, Japan) using about 100 ng cDNA as the template. All procedures were performed according to the manufacturer’s instructions. PCR was conducted using a CFX96 real-time system (Bio-Rad, Hercules, CA, United States) with PCR conditions of 95°C for 5 min, then 40 cycles at 95°C for 5 s and 60°C for 30 s, and an extension at 72°C for 10 min, same as our previous study (You et al., 2017). Data was normalized by the level of housekeeping gene *sigA* in each individual sample. The relative transcript levels of target gene were calculated using the 2^–ΔΔCt^ method.

### Determination of Affinity Constant (KD Value)

The binding affinities of GlnR and PrpR proteins to the upstream region of the icl operon were determined by Bio-Layer interferometry using an Octet System (Octet QKe; ForteBio, United States). Streptavidin biosensors were loaded with biotinylated DNA fragment (upstream region of *icl*) by incubation for 5 min in 7 μg/ml DNA solution (containing 10 mM HEPES, 2 mM magnesium chloride, 0.1 mM EDTA, 200 mM potassium chloride, pH 8.0), and then washed in loading buffer for 5 min. After that, the biosensors were moved to protein solutions to allow association for 10 min, and then transferred into running buffer (containing 10 mM HEPES, 2 mM magnesium chloride, 0.1 mM EDTA, 200 mM potassium chloride, 10 μg/ml bovine serum albumin, 0.02% Tween-20, pH 8.0) to detect dissociation. All incubations were performed in a 96-well plate at 37°C at 1,000 rpm in a volume of 100 μL. The kinetic parameters K_on_, K_off_, and KD were calculated by 1:1 binding model using the Octet Data Analysis version 7.0.

### Scanning Electron Microscopy (SEM) Analysis

Strains were cultivated to log phase and harvested in 4°C with centrifuge of 6000 × *g*, washed twice and resuspended with normal saline. Strains were stained with 2% phosphotungstic acid and after 2 min later, analyzed the morphology of bacterial by JEM-4100. Length of bacteria was analyzed by manual evaluation, using Image J on the electron microscopic images.

### Liquid Chromatography-Mass Spectrometry (LC-MS) Analysis

Bacteria were cultured to log phase in Sauton’s medium with 10 mM sodium propionate under the N^L^ (1 mM ammonium sulfate) or the N^XS^ (30 mM ammonium sulfate) conditions, collected by centrifugation and then frozen in liquid nitrogen for 1 min. After being restored to the room temperature, bacteria cells were treated with 75% boiling ethanol, heated in boiling water for 5 min, and then frozen at −80°C. Followed by high speed centrifugation, supernatants containing the intracellular organic acids were collected. Samples were subsequently concentrated by vacuum concentrator for LC-MS detection. Metabolites were separated on a ZORBAX Eclipse XDB-C18 column (4.6 × 250 mm, 5 μm) at 30°C, the mass spectrometer used was an Agilent Accurate Mass 6530 Q-TOF coupled to an Agilent 1260 LC system. For elution, 1‰ (v/v) methanol (solvent A) and acetonitrile (solvent B) were used as the mobile phases at a flow rate of 1 mL/min. Data was analyzed by the “Q-TOF Quantitative Analysis.” Calibration curves were performed by serial dilutions for different organic acid’s standards to test correlation between chromatogram peaks areas and analyte quantities. All samples were analyzed in triplicate and results were reported as the average with standard deviation.

### Cell Culture, Infection and Assessment of Cell Death

THP-1 cells were grown in RPMI 1640 (Gibco) supplemented with 10% fetal bovine serum (FBS; Gibco), 1% penicillin and streptomycin (Gibco). The cells were seeded onto 12-well culture dishes at a density of 2 × 105 cells ml^–1^ and treated overnight with 10 ng/ml phorbol myristate acetate (Sigma). Cells were washed three times with phosphate buffer saline and incubated for one more day, then infected with Msm wild type, Δ*glnR*, Δ*glnR*::*glnR* strains (killed bacteria as control) for 2 h with MOI at 10, 5, and 1 (strains were washed three times with normal saline). The infected cells were washed three times with RPMI 1640 medium to eliminate the suspended bacteria and then incubated in fresh RPMI 1640 medium with 15 μg/ml gentamicin for 72 h. Annexin V-FITC&PI Apoptosis Detection Kit (Sangon Biotech, China) was used according to the protocol in order to mark apoptotic THP-1 cells. Stained cells were analyzed immediately on CytoFlex (BECKMAN COULTER) and further analyzed with CytExpert.

### MTT Assay

THP-1 cells (5 × 10^4^ cells ml^–1^) were seeded in a 96-well plate in triplicate and incubated for 12 h for cell attachment, replace a new medium, after 12 h added the *Msm* wild type, Δ*glnR*, Δ*glnR*::*glnR* strains with MOI at 10,5,1 into the 96-well plate. At 72 h post-infection, MTT [3-(4,5-Dimethylthiazol-2-yl)-2,5-Diphenyltetrazolium Bromide] solution was added to the 96-well plate and incubated for 4 h at 37°C, add the Formazan solution slowly and then continue incubation about 4 h. OD_570_ values were measured in a microplate reader (Synergy H1, Biotek).

### Survival of *Msm* in Macrophages

THP-1 cells were divided into 12-well plates (2.0 × 10^5^ cells ml^–1^) and differentiated using phorbol myristate acetate when the cells were at the best state (He et al., 2017). After 12 h, the cells were washed and then cultured in fresh RPMI 1640 medium for 12 h. The *Msm* wild type, Δ*glnR*, Δ*glnR*::*glnR* strains were added into the plates (10 times more than the macrophage cells), with MOI at 10, 5, 1. After incubating for 2 h, the cells were washed three times using fresh medium to remove the uninfected bacteria. The infected cells were cultured in fresh medium with gentamicin. After 72 h, the cells were washed three times with fresh medium, and then LB medium containing 0.05% SDS was added to lyse the cells for 10 min. The lysates were collected and diluted at different gradients to the inoculate plate. The *Msm* colony-forming units were counted after 3 days of culturing (Tiffert et al., 2008).

## Data Availability Statement

The original data presented in the study are included in the article/Supplementary Material, further inquiries can be directed to the corresponding author/s.

## Author Contributions

NQ and G-LS was responsible for experimental design, data collection, and writing manuscript. WD participated in plasmid construction and HPLC analysis. B-CY was responsible for experimental design, data analysis, and final approval of the manuscript. All authors contributed to the article and approved the submitted version.

## Conflict of Interest

The authors declare that the research was conducted in the absence of any commercial or financial relationships that could be construed as a potential conflict of interest.

## Footnotes

1 http://www.ncbi.nlm.nih.gov/pmc/articles/PMC3245079/
