# Supplementary material for: Mycobacterium smegmatis GlnR Regulates the Glyoxylate Cycle and the Methylcitrate Cycle on Fatty Acid Metabolism by Repressing icl Transcription
Source: Front Microbiol. 2021 Feb 3;12:603835. doi: 10.3389/fmicb.2021.603835 (PMC7886694; doi:10.3389/fmicb.2021.603835)
Supplement: Supplementary file 1 [file Data_Sheet_1.PDF]

Supporting Information

Mycobacterium smegmatis GlnR regulates the glyoxylate cycle and the methylcitrate cycle on fatty acid metabolism by repressing *icl* transcription

Nan Qi, Guo-Lan She, Wei Du, Bang-Ce Ye

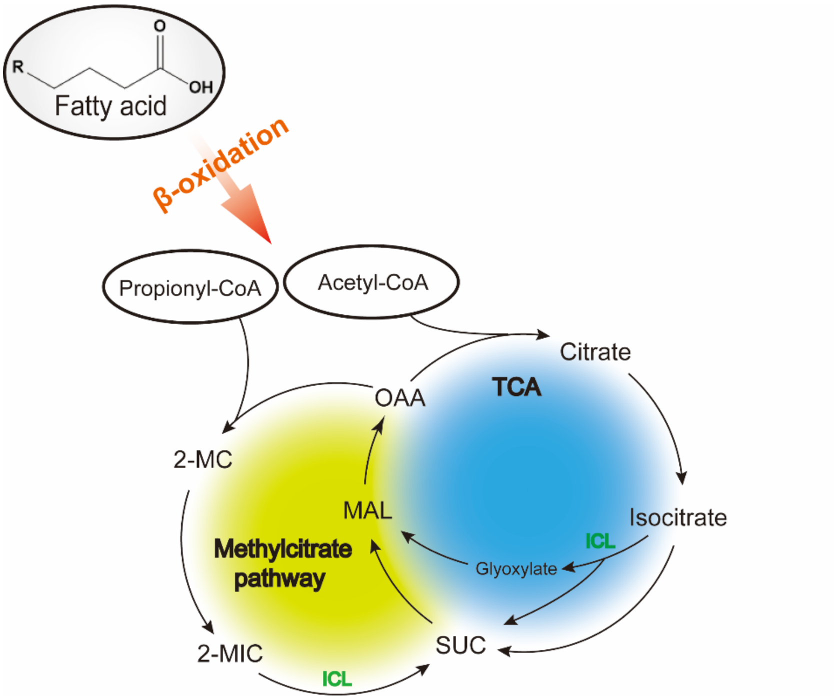

Figure S1. A model illustrating the dual-role of ICL in the metabolism of fatty acids in *Msm* through the glyoxylate cycle and the methylcitrate cycle. Circle with blue: TCA cycle and glyoxylate cycle; circle with yellow: Methylcitrate cycle; SUC, succinate; MAL, malate; OAA, oxaloacetate; 2-MC, 2-methylcitrate; 2-MIC,2-methylisocitrate; ICL, isocitrate lyase.

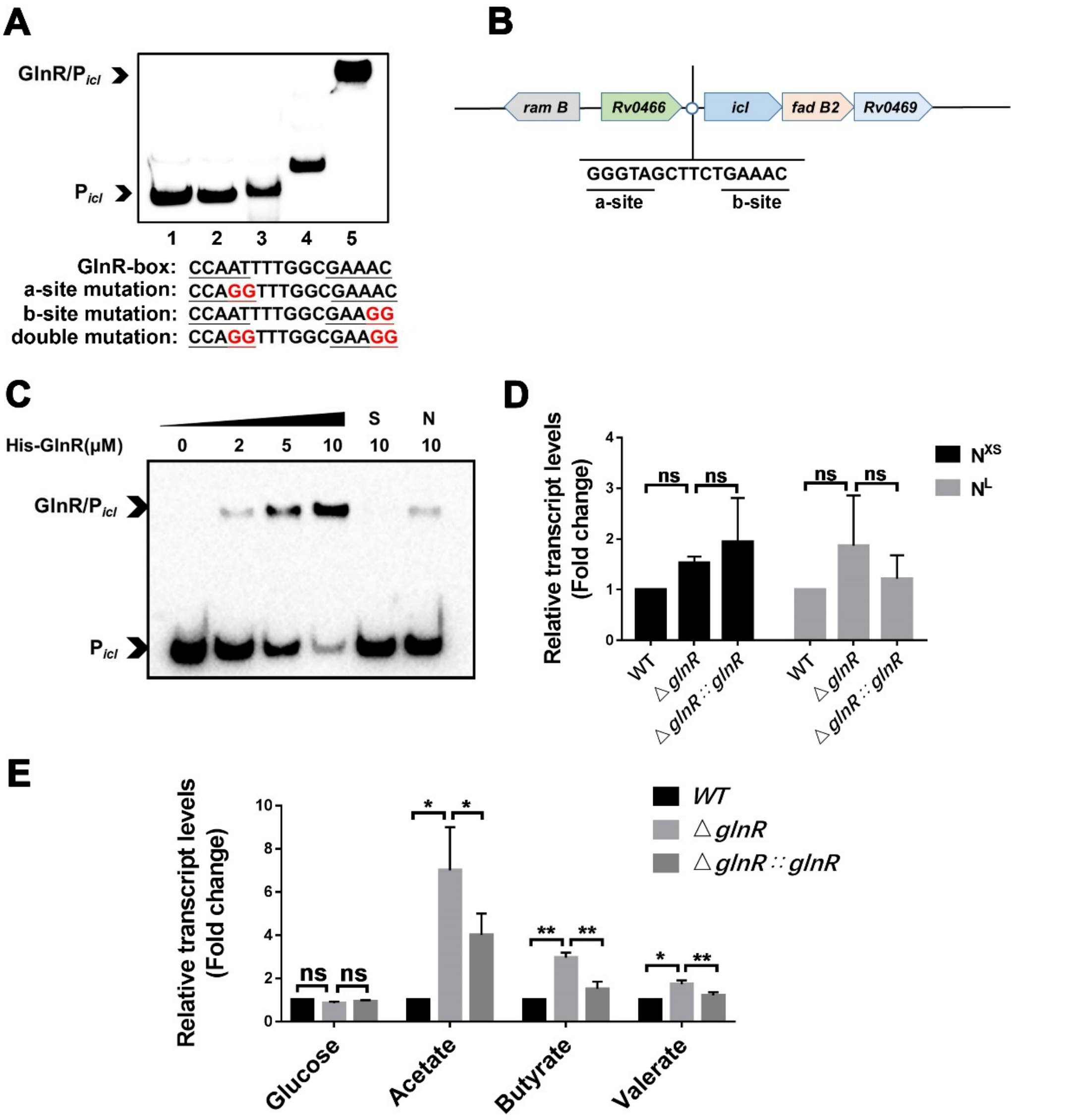

Figure S2. Mutagenesis analysis of the GlnR-box in the promoter of *icl* (A) EMSA showing the binding activity of GlnR with WT and mutated GlnR-box in the *icl* promoter of *Msm*. Experiment was conducted as described in Figure 1B. 2 ng WT and mutated P*icl* probes derived from GlnR-box were incubated with 0.5 μM recombinant His-GlnR proteins. The a-site and b-site in the GlnR-box were indicated by underlined sequences, in which the AT to GG and AC to GG mutations were marked in red. Lane 1, free WT P*icl* probe (-CCAAT-n6-GAAAC-).; lane 2, double site-mutated P*icl* probe (-CCA**GG**-n6-GAA**GG**-) with GlnR; lane 3, b-site-mutated P*icl* probe (-CCAAT-n6-GAA**GG**-) with GlnR; lane 4, a-site-mutated P*icl* probe

(-CCA**GG**-n6-GAAAC-) with GlnR; lane 5, WT P_{icl} probe with GlnR. (B) A diagram illustrating the putative GlnR-binding motif in the *icl* promoter of *Mtb*. (C) EMSA showing the binding of recombinant GlnR with the upstream promoter region of *icl* in *Mtb*. The experiment was conducted as described in Figure 1B. The DNA probe P_{icl} (2 ng in a 10 μL reaction system) was incubated with a concentration gradient (0, 2, 5, and 10 μM) of His-tagged GlnR. S represents the unlabeled specific probe, N represents the non-specific competitor DNA (Salmon sperm DNA). (D) The transcript levels of *MSMEG_3706* in wild type (WT), Δ*glnR* and Δ*glnR::glnR* strains of *Msm* under different concentrations of nitrogen sources. The *Msm* wild type, Δ*glnR* and Δ*glnR::glnR* strains were cultivated in the nitrogen-limited (N^L) and nitrogen-excess (N^{XS}) media and collected at the log phase. RNA was extracted and qRT-PCR was performed to determine the relative transcript fold (i.e. mutant vs WT) of *MSMEG_3706* using the $2^{-\Delta\Delta Ct}$ method. (E) The transcript levels of *icl* in wild type (WT), Δ*glnR* and Δ*glnR::glnR* strains of *Msm* growing on a variety of carbon sources under the nitrogen starvation condition. The *Msm* wild type (WT), Δ*glnR* and Δ*glnR::glnR* strains were cultivated in the indicated carbon sources and collected at the log phase. RNA was extracted and qRT-PCR was performed to measure the relative transcript fold (i.e. mutant vs WT) of *icl*. Data are presented as mean values with error bars indicating standard deviations (±SD) calculated from three independent experiments. Unpaired two-tailed Student's t test, *P<0.05, **P<0.01, ns indicates no statistically significant difference.

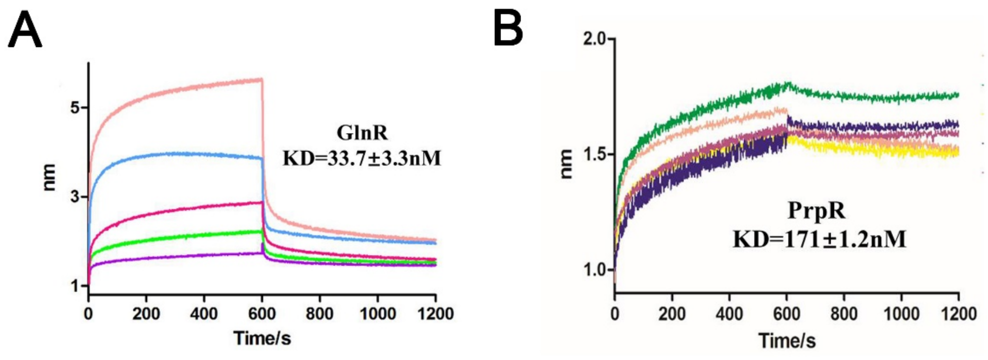

Figure S3. GlnR has a higher affinity for the *icl* promoter than PrpR.

(A-B) Octet assays of GlnR-*icl* and PrpR-*icl*. Binding of biotin-labeled probe P_{icl} with increasing concentration of His-GlnR (A; 1.2, 0.9, 0.6, 0.3, and 0.015 μM) and His-PrpR (B; 4.8, 2.4, 1.6, 1.2, 0.6 μM). The KD value of GlnR and PrpR is 33.7±3.3 nM and 171±1.2 nM, respectively.

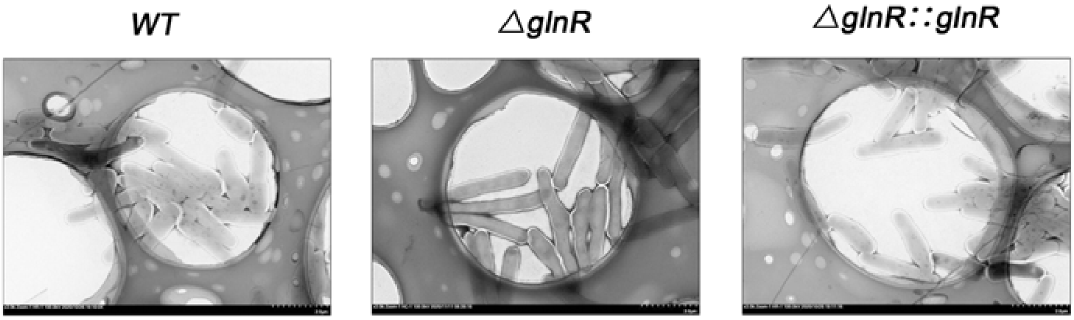

Figure S4. Scanning electron microscopy images of bacteria cells from diverse *Msm* strains growing on propionate. Experiment was conducted as described in Figure 4D. Length of bacteria cells was measured by manual evaluation, using Image J on the electron microscopic images.

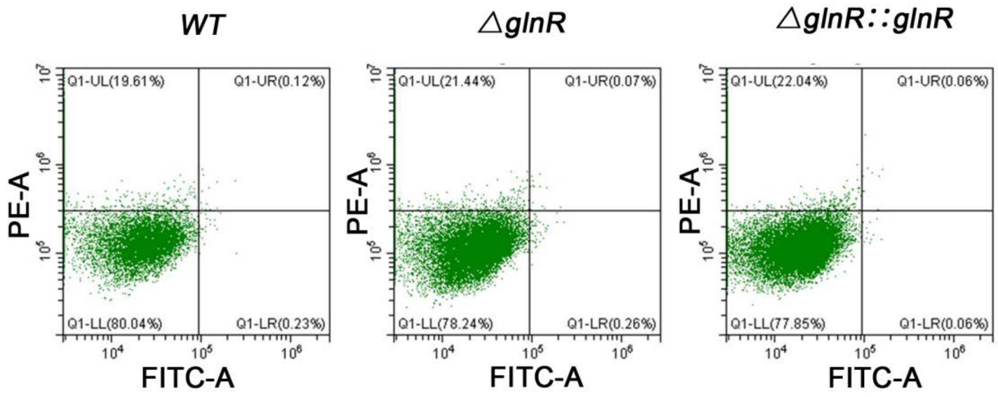

Figure S5. The apoptosis of macrophage induced by *Msm* is not related to metabolites. The apoptosis of macrophages infected with killed bacteria. After killed bacterial infection of macrophages for 72h. The effects of *Msm* WT, Δ*glnR*, and Δ*glnR::glnR* strains on cell apoptosis were determined through the FCM. Lower right (LR) represents the apoptotic cells and upper right (UR) represents the necrotic cells.
